# Peer group supervision is an efficient method to assist midwives to solve complicated cases: A case-study in the Women’s Clinic, East-Tallinn Central Hospital, Estonia

**DOI:** 10.18332/ejm/112255

**Published:** 2019-09-30

**Authors:** Marika Merits, Irena Bartels, Annely Kärema

**Affiliations:** 1 Midwifery Department, Health Education Centre, Tallinn Health Care College, Tallinn, Estonia; 2 East-Tallinn Central Hospital Women’s Clinic, Tallinn, Estonia

**Keywords:** peer group supervision, midwives’ activities, complicated cases

## Abstract

**INTRODUCTION:**

Midwives have a remarkable influence on various outcomes in healthcare, of which the activities related to the management during the childbirth are emphasised the most. Midwives like all healthcare workers encounter many different stressors in clinical practice, including time pressure, excessive workload, different roles, and emotional issues. The profession of the midwife requires much knowledge, competence, good attitude and self-confidence in order to cope with many different complicated situations and dilemmas. This study has been conducted under the project: ‘Increasing midwives’ ethical competence: a European educational and practice development project (INEC)’.

**METHODS:**

This is a qualitative case study. The current case study involved 7 midwives of the Women’s Clinic, East-Tallinn Central Hospital, Estonia; the midwives expressed their willingness and agreed to take part in the activities of the peer group supervisors. The aim of the case study was to find how peer group supervision as a method helps midwives to solve complicated cases and dilemmas, helping them to maintain self-confidence and therefore to manage stress at work more effectively.

**RESULTS:**

All the participating midwives shared the opinion that peer group supervision helps midwives to cope with complicated situations and dilemmas more effectively, it empowers professional skills and self-confidence, and so contributes to more effective stress management at work. Also, the midwives expressed hope that this topic would be useful for the professional midwives and to other employees working in the field of healthcare, that encounter complicated cases in their daily work, and so will form an active team of peer group supervision.

**CONCLUSIONS:**

Peer group supervision is an efficient method that helps midwives solve complicated cases in the Women’s Clinic, East-Tallinn Central Hospital, Estonia.

## INTRODUCTION

Midwives have a remarkable influence on various outcomes in healthcare, of which the activities related to the management of the process of delivery are emphasised the most^1-3^. The profession of the midwife requires much knowledge, competence, experience and self-confidence in order to cope with many different complicated situations and dilemmas^4^. It has been acknowledged worldwide that the competent activity of the midwife has a positive influence on the health of the mothers and the newborns, and midwives contribute in the best possible way to achieve this. Midwives offer competent and culturally sensitive care and are thus respected by society. In reality, these skilled and caring midwives offer more than ensuring that a child is born in a safe way^5^. Midwives like all healthcare workers encounter many different stressors in clinical practice, including time pressure, excessive workload, different roles, and emotional issues^6^. Management of complicated cases and common work-related stress influence the physical and mental well-being of midwives that may result in ‘burn-out’ and/or traumatic symptoms. These phenomena not only influence midwives’ well-being and health, they also damage professional capacity and the quality of care provided^7,8^. Many studies confirm that the work of the midwife is emotionally very demanding^9-12^. Caring about the women and their families requires coping with different psychological tensions by the midwives such as anxiety, pain, fear, even sometimes death and grief, but also excitement and happiness. While working in these emotionally intense situations, the personal wholeness of midwives often remains unpreserved or is undervalued^13,14^. Caring and empathy are important components in the activities of midwives, but encountering traumatic events, diseases or death in their daily work can cause compassion fatigue. Compassion fatigue is a state caused by the surfeit of patients’ suffering, characterised by inability and incapability to feel compassion for the people in need, to help them and to adequately evaluate their condition^10,15^. Therefore, the midwives absolutely need an efficient method to find solutions to complicated cases, to improve their professional skills and manage work-related stress. One such possibility in the activities of the midwife is the method of peer group supervision where the members of staff equally doing similar work support each other, which in the current context are the midwives^5,16-19^. Peer group supervision is also defined as a structured examination of cases in the group of colleagues or the specialists connected by the case. Peer group supervision is conducted by the appropriately skilled head of peer group supervision, an employee authorised by the staff (the midwives)^20^. The team of peer group supervision acts with agreed regularity with the aim of finding workable solutions to difficult work and/or client cases. The structured method helps to keep in focus both the cases and the professional skills, in order to solve complicated situations and ethical dilemmas^21,22^.

## METHODS

This study has been conducted under the project: ‘Increasing midwives’ ethical competence: a European Educational and Practice Development project (INEC)’. The current project was initiated to develop a set of interventions in the clinical and educational setting in order to create a model for education and practice that would increase the ethical competence of midwives facing today’s clinical challenges^23^. It is a case study focused on seven midwives that expressed their wish to participate voluntarily in the activities of a team of peer group supervision. The aim of this case study in Estonia was to ascertain how peer group supervision as a method helps midwives to solve complicated cases and dilemmas, helping them to stay self-confident and thereby manage stress at work more effectively. The study received ethical approval and is in accordance with the ethical grounds of the INEC project. The study followed the ethical guidelines of the Women’s Clinic, Estonian East-Tallinn Central Hospital, and was approved by the respective authorities. Participation in the study was voluntary.

The aim of the case study, the method, and the process of peer group supervision were explained to the participating midwives. Also, the importance of security and confidentiality were explained (discussions and information remaining within the boundaries of the group), as well as mutual understanding and support, suggestions, constructive advice, and sharing professional knowledge and skills. The peer group supervision team was active for 8 months, from October 2015 to May 2016. The meetings took place once a month and each meeting lasted approximately 1.5 hours. The meeting room was not located in the Women’s Clinic, was cosy and offered a safe environment for the participating midwives. Professional work experience of the participating midwives varied from 3 to 24 years. The youngest participant was aged 28 years and the eldest 50 years.

At their first meeting, the theoretical approaches of peer group supervision, the possibilities and methods, and the difference between supervision and job coaching and peer group supervision were introduced to the midwives. As an essential part, the nature of the role of the head of the peer group supervision was explained, which included process management, time tracking, and summarization.

## RESULTS

During their first meeting various factors causing stress during midwives work were discussed under the guidance of the head. The participating midwives mentioned several stress factors in their work such as time pressure, excessive workload, compassion fatigue and/or mental fatigue, and conflicts etc. The participating midwives considered complicated cases related to patients’ health and wellbeing the most crucial. Thus, the main topic in peer group supervision is solving the work-related complicated cases, how to maintain self-confidence and manage stress at work more effectively. The participating midwives shared the opinion that several cases must be strictly midwifery-related, actual, topical and complicated, already happened or happening in the near future. Also, it was agreed that cases from work practice should be brought to the meetings and one chosen for discussion.

The participating midwives were asked to evaluate subjectively how they cope with stress at work when solving complicated cases, this was asked both at their first and last meeting, and they were requested to highlight if something had changed.

Within the current case study, the phases of the method of peer group supervision that form the basis for solving a case were explained to the midwives.

During the *introductory* phase, helpful exercises were conducted for creating a tension-free environment and enabling ‘tuning-in’, after that, the owner of the case (in this case a midwife) gave an outline of the case to the other members of the group. The introductory phase was followed by five phases:

*Phase 1* – the task of the midwives was only to listen to the case quietly and get a feel for it, but not to ask questions.

*Phase 2* – so-called detailed questions or ‘dark area’ questions were asked. According to Egan^24^ ‘dark area’ questions are those that give more necessary information about the case and help in the process of finding a solution later. ‘The owner of the topic’ answered the questions. Only detailed questions were asked; the topic itself was not elaborated. When a question was asked, the owner of the case did not have to answer the same question twice. This phase also followed the rule of not giving any recommendations or suggestions.

*Phase 3* – the midwives analysed the case between themselves, whereby ‘the owner of the topic’ did not get involved in the discussion. The aim of this phase was to deepen understanding and empathy, and prepare them for finding suggestions.

*Phase 4* – the midwives gave suggestions to the owner of the case (also to colleagues related to the case). Every midwife gave one to two suggestions. If required, the suggestions were given in a written form. In this phase, the midwives gave suggestions to ‘the owner of the topic’ who did not comment on each separately. After having received the suggestions from all the members of the group, they shared which method or suggestion was considered most useful and what could be implemented in the real activities of the midwife. This phase is also called a consultation phase.

*Phase 5* – in this conclusion phase, the midwives briefly shared what they had learned from this case, mentioning the several dilemmas that occurred and recognising that sometimes there are not only ‘positive options’ available. The opinion was shared that in a complicated case there must be collegial preparedness and options to help the colleague during the process of the case, there must be understanding, sharing of responsibility and emotions, giving professional advice verbally, and providing supporting actions, all of which play important roles. In this last phase, thanking each other and maintaining a positive background play also important roles. The head of the peer group supervision team helps with summarising and emphasising important moments.

During the case study lasting 8 months overall, the cases presented by all the participants were solved, the phases of peer group supervision were practised and the beneficial factors of all the solved cases for the participants were analysed.

## DISCUSSION

A number of applications of peer groups have been described in midwifery and nursing literature, encompassing both clinical and academic settings. Current peer group supervision used a method of 5 phases and a process for solving a complicated case based on similar approaches by Aava^19^ and Tietze^21,22^, which have a simple structure and are well-defined. Based on the literature, there is no single effective peer group supervision method and/or process being used in healthcare institutions. Several studies have described various methods of supervision, process and its elements. Begat et al.^25^ used a method in which peer group supervision was combined with education in nursing science inspired by a holistic nursing ‘model’; De Graaff and Francke^26^ used a 3-step model, concentrating on norms and values of solving a case; while Häggström et al.^27^ combined lectures and the peer group supervision method for empowering the activities of healthcare employees.

This case study showed that in the team of peer group supervision it is difficult for the midwives to start their activity immediately with solving a case (it may be emotionally traumatic). Therefore, it is advisable to use preparation and ‘tuning-in’ themselves beforehand to create a tension-free atmosphere and background, as necessary as possible, during the discussion of complicated cases and dilemmas. There are many options/methods for ‘tuning-in’ oneself. The participating midwives were introduced to several creative settings methods, canalisation and specific ‘tuning-in’ exercises, suitable for the beginning of group work. At first, the sensation of anxiety was experienced that caused resistance or the wish not to take part in the exercises, with examples for an excuse being: *‘I look weird to the others’ or ‘What must they think of me?’* etc. Insecurity and fear can greatly stifle creativity. One of the introductory ‘tuning-in’ exercises was the compliment exercise, where the clinic’s midwives wrote something positive on separate pieces of paper to all members of the group, characterising briefly the professional skills and personality traits of their colleagues. The exercise received positive feedback, lessened anxiety, and designed the necessary preparation for solving a case. The midwives shared with each other the emotions that the compliment exercise produced: *‘I could not even guess that I am considered as a competent and a friendly colleague’, ‘My heart softened reading all these pleasant comments about me’, ‘When I am having a difficult day or I am in a bad mood, then I read what the colleagues have written about me’, ‘I am flattered’* etc. The exercises empowered collegiality and confirmed the knowledge that they were interconnected members of a team. Thus, every time the group of midwives met, some suitable ‘tuning-in’ or creative exercise was implemented.

Several studies confirm that it is imperative to talk about and/or analyse the feelings of a healthcare employee, including a midwife. It allows one to understand oneself better, to maintain or restore self-confidence, and it is a significant component when finding solutions to cases. Berg et al.^28^ and Berg & Hallberg^29^ emphasise the importance of describing and analysing feelings, especially during the first meeting of peer group supervision. It creates an environment without tensions, allowing one to understand and analyse the patient’s feelings too.

During the first meetings, there were difficulties in following the rules of the phases of peer group supervision by the midwives. For instance, giving advice or explaining the details were desired immediately after the presentation of a case description. In that situation, the head of peer group supervision had to explain, additionally, why following the rules of the peer group supervision phases is important, since every rule has an empowering force. Also, following the rules helps to manage the immediate emotions of the participants, reflect the meaningful emotions, convey an argument’s point of view, and give constructive advice. The midwives’ awareness of the process of peer group supervision and the importance of rules were deepened from the third meeting. During the last of the 8 meetings, the midwives emphasised that they had acquired the principles of solving complicated cases, and it was noted that the more complicated the discussed case in peer group supervision was, the more time it took to discuss it between them and for empowering each other.

When analysing the contents of the cases, the participating midwives’ discussion was focused on patients’ health and wellbeing, by describing the communication as well as quality and/or problems of clinical skills. For instance, the Engels et al.^30^ study focussed on peer group supervision on improving the quality of clinical cases only in the activities of the midwife. On the other hand, Cheyne et al.^31^ assessed the whole process of peer group supervision in the work of a midwife. Thus, they did not use a single method when discussing a case and focussed on different aspects of a case.

In addition, the midwives emphasised that during the 8 months in which the peer group supervision took place, the collegiality between them improved, while the capability to share responsibilities and readiness to solve complicated cases and dilemmas, work satisfaction and the joy of working increased. Brodie5, Bäck et al.^3^, and Lavery et al.^18^ confirmed a similar point of view. It is emphasised that peer group supervision helps to empower trust between midwives and also improves the resilience and capability to develop in different professional fields. Peer group supervision allows the midwives to experience mutual connection such as similarity of cases and the feeling of being supported, and provides them with the knowledge that they are valued.

The participating midwives were asked to assess subjectively how they manage stress at work when finding solutions to complicated cases both at their first meeting and at their last meeting, and to indicate the kind of changes that occurred. When they first met, the midwives mentioned different factors that cause stress at work such as time pressure, excessive workload, compassion fatigue and/or mental fatigue, conflicts at work etc. The participating midwives thought that the most important were problems that arose in solving complicated cases, related to patients’ health and wellbeing, and how to maintain self-confidence to cope with stress at work more effectively. The midwives noted during the last meeting that time pressure and excessive workload had remained the same, but these were more emphasised as natural parts of a midwife’s work. There was an improvement in skills dealing with cases and in self-confidence when solving them and in the collegiality when supporting each other at work.

### Strengths and limitations

Implementing a peer group supervision method has both strengths and limitations. The strengths include: the high motivation of the participating midwives and personal contribution during the period of participation at peer group supervision; compatibility between people and minor instances of discrepancy; the courage to listen and share emotions and professional experience; and readiness to continue activities of peer group supervision in the future. The limitations of the peer group supervision method include: more complicated cases require more time and are therefore particularly tiring; it is difficult to accept the absence of right solutions; it is challenging to plan a common appropriate peer group supervision time for all members; all activities of peer group supervision have been presented only by an example of a single group of 7 midwives, but in order to improve the credibility of the method it is essential to have more groups and/or participants to measure the effectiveness of the method using a quantitative method; management of stress at work when finding solutions to complicated cases was measured subjectively only at the first and last meeting, however to improve the credibility it would be necessary to use various metrics.

## CONCLUSIONS

The current case study confirmed that peer group supervision is an efficient method in solving complicated cases and dilemmas in the activities of midwives. When implemented on a regular basis, it is possible to more efficiently cope with different work-related tensions and difficult situations.

All the participating midwives shared the opinion that peer group supervision helps one to cope with complicated situations and dilemmas better; it also empowers professional skills and self-confidence. Also, midwives expressed hope that this topic would be useful for the working midwives and other employees working in the field of healthcare that encounter complicated cases in their daily work, who they hoped will participate in a team of peer group supervision. The activity of peer group supervision helps midwives to understand each other better in complicated and difficult situations and to learn how to support colleagues. We agree with the views of the participants and also express hope and belief that this topic becomes relevant for the working midwives and to others working in the field of healthcare that meet complicated cases in their daily work and it is hoped that they will form a team of peer group supervision in their institution.
